# Supplementary figures and images for: Selective YAP activation in Procr cells is essential for ovarian stem/progenitor expansion and epithelium repair
Source: eLife. 2022 Mar 14;11:e75449. doi: 10.7554/eLife.75449 (PMC8920503; doi:10.7554/eLife.75449)

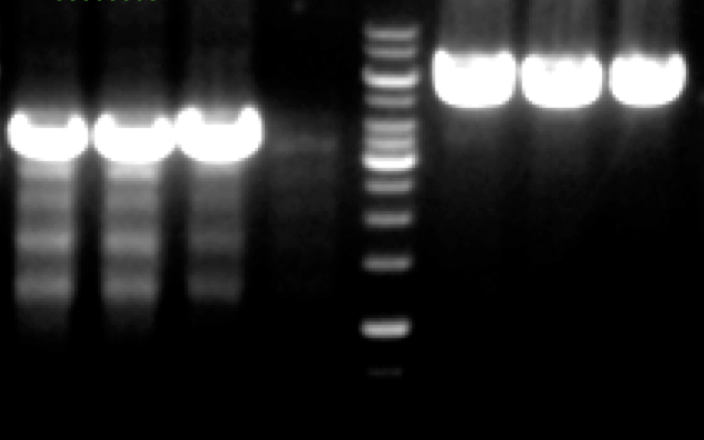

Supplement: Figure 3—figure supplement 1—source data 2. [file elife-75449-fig3-figsupp1-data2.zip › Figure 3-figure supplement 1-Panel B-source data 1.png]

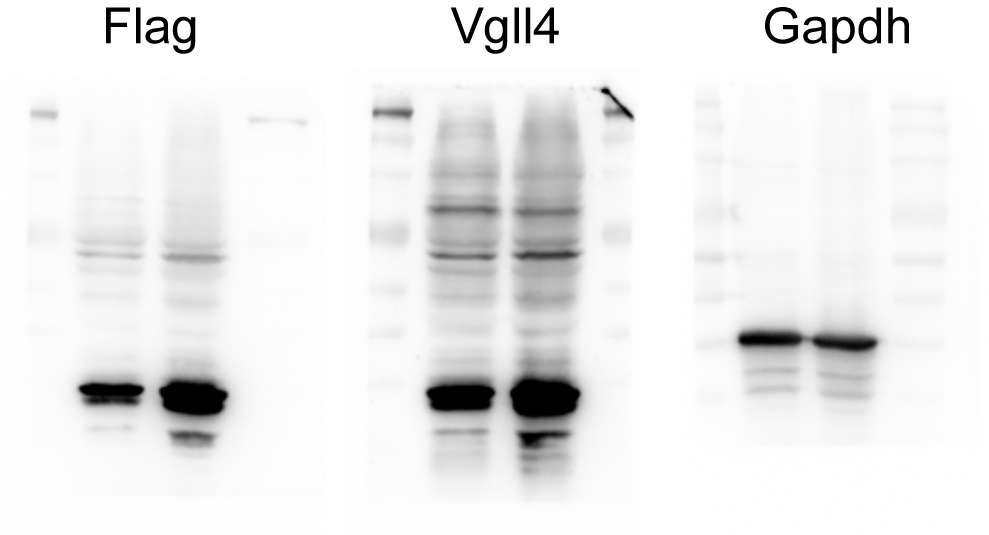

Supplement: Figure 3—figure supplement 1—source data 2. [file elife-75449-fig3-figsupp1-data2.zip › Figure 3-figure supplement 1-Panel D-source data 1.tif]

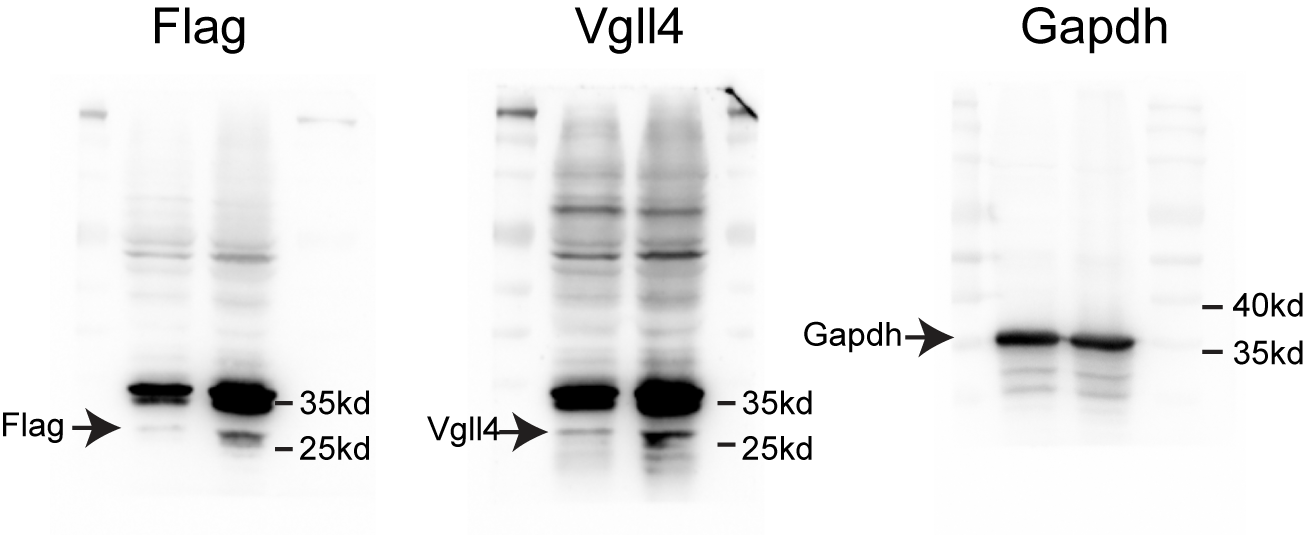

Supplement: Figure 3—figure supplement 1—source data 2. [file elife-75449-fig3-figsupp1-data2.zip › Figure 3-figure supplement 1-Panel D-source data 2.tif]

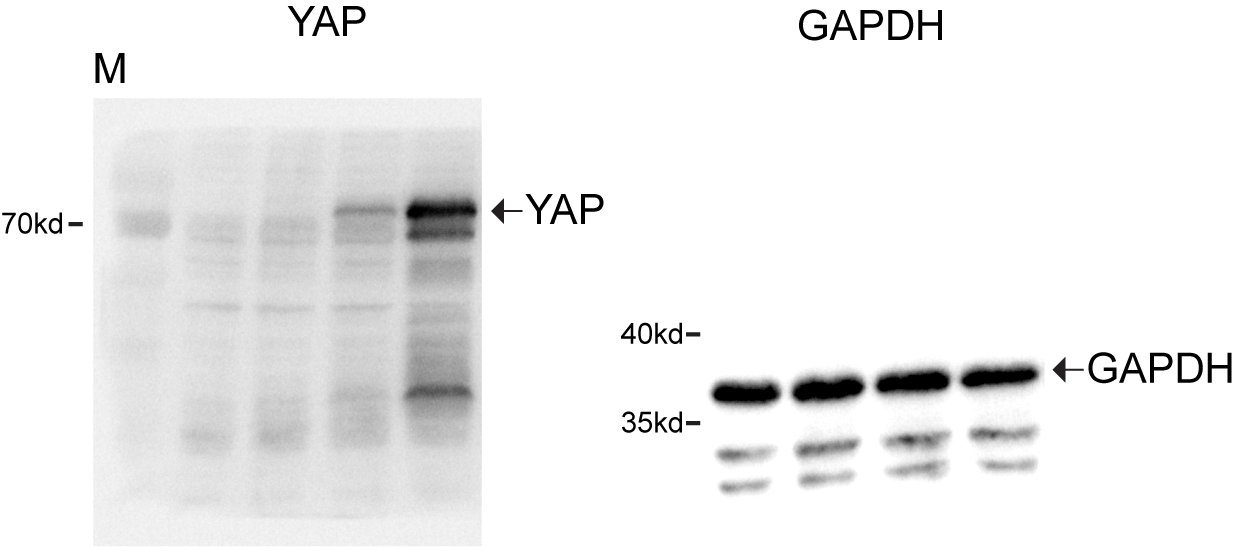

Supplement: Figure 4—figure supplement 2—source data 2. [file elife-75449-fig4-figsupp2-data2.zip › Source data-Figure 4-figure supplement 2-2.tif]
